# Supplementary material for: Mechanism of Curcuma wenyujin Rhizoma on Acute Blood Stasis in Rats Based on a UPLC-Q/TOF-MS Metabolomics and Network Approach
Source: Molecules. 2018 Dec 27;24(1):82. doi: 10.3390/molecules24010082 (PMC6337646; doi:10.3390/molecules24010082)
Supplement: Supplementary file 1 [file molecules-24-00082-s001.pdf]

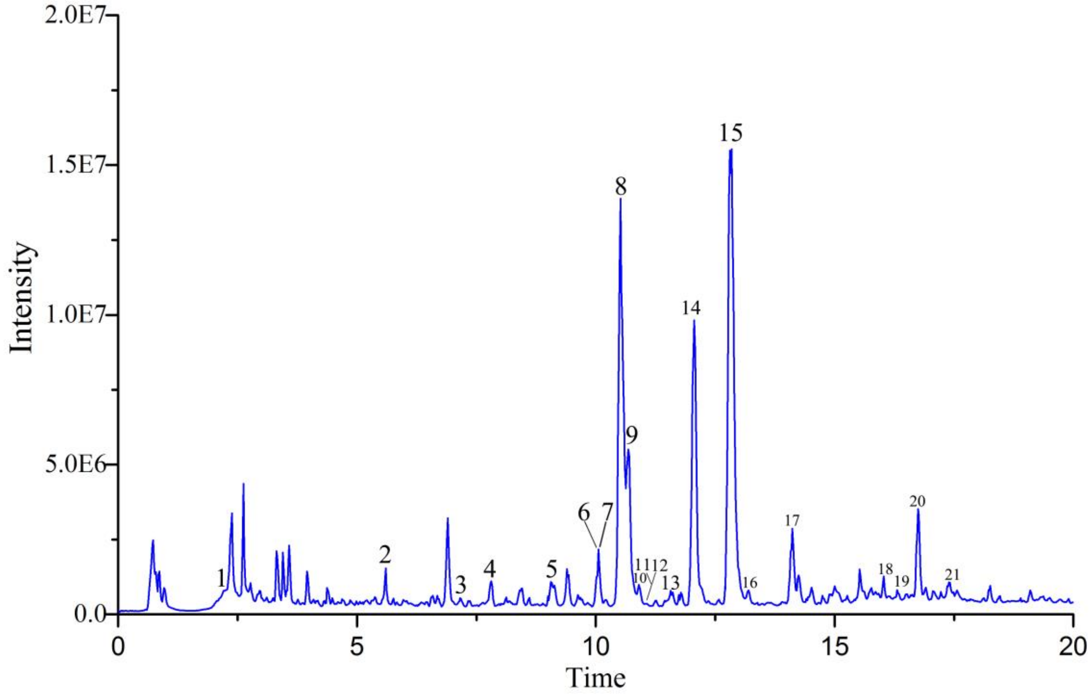

Fig. S1 Total ionic chromatogram of EZhu by UPLC-Q/TOF-MS.

Table.S1 Identification of 21 chemical compounds in EZhu by UPLC-Q/TOF-MS

No.	RT (min)	Formula	M(Da)	$[M+H]^+$（m/z）				
				Indicated	ppm	Prominent MS^2 fragmentation	Identification	metabolite class
1	2.16	$C_{15}H_{20}O_5$	280.1310	281.1389	1.8	281,245,227,203,156,107,91	Zedoalactone B	guaiane-type
2	5.74	$C_{15}H_{22}O_3$	250.1569	251.1644	1.1	233,215,191,173,165,159,145	Aerugidiol	guaiane-type
3	7.06	$C_{15}H_{20}O_4$	264.1362	265.1434	2.5	247,229,121,105,91	Zedoarofuran	eudesmane-type
4	7.51	$C_{15}H_{18}O_3$	246.1256	247.1333	1.5	247,229,189,183,128,105	Zederone	germacrane-type
5	9.16	$C_{15}H_{24}O_3$	252.1725	253.1800	0.6	253,235,189,175,147,133,107	Zedoarondiol	guaiane-type
6	10.25	$C_{15}H_{20}O$	216.1514	217.1588	0.3	217,199,169,157,128,131,55	Furanodiene	germacrane-type
7	10.25	$C_{15}H_{22}O_2$	234.1620	235.1693	0.3	235,217,189,135	Curcumenol	guaiane-type
8	10.71	$C_{15}H_{22}O_2$	234.1620	235.1693	0.5	235,177,161,105,91	Curcumenone	carane-type
9	11.15	$C_{15}H_{16}O$	212.1201	213.1274	-0.2	213,197,155,128,69	Pyrocurzerenone	laserane-type
10	11.17	$C_{15}H_{18}O_2$	230.1307	231.1381	-0.2	231,213,185,157,142,128	Curzerenone	elemane-type
11	11.20	$C_{21}H_{22}O_6$	370.1416	371.1489	0.5	371,177,137,117	Dihydrocurcumin	curcumins-type
12*	11.23	$C_{20}H_{18}O_5$	338.3539	339.1230	1.0	339,255,223,177,147,119	Demethoxycurcumin	curcumins-type

13*	11.57	$C_{21}H_{20}O_6$	368.1260	369.1339	1.0	369,285,253,177,145,117	Curcumin	curcumins-type
14	12.25	$C_{15}H_{24}O_2$	236.1776	237.1851	0.9	237,219,135,107,93,81	Neocurdione	germacrane-type
15*	13.03	$C_{15}H_{24}O_2$	236.1776	237.1848	-0.5	219,135,107,93,79,67	Curdione	germacrane-type
16	13.47	$C_{15}H_{16}O_2$	228.1150	229.1223	2.1	229,213,201	Curzeone	cadinane-type
17	14.37	$C_{15}H_{20}O_2$	232.1463	233.1536	0.8	233,175,147,105,119,91	Furanogermenone	germacrane-type
18	16.38	$C_{15}H_{20}O_3$	248.1410	249.1488	1.0	249,203,163,143,105,91,69	Curcumenolactones A	carane-type
19*	16.55	$C_{15}H_{24}O_2$	236.1776	237.1850	0.5	237,163,135,95,69	Curcumol	guaiane-type
20*	17.02	$C_{15}H_{22}O$	218.1671	219.1745	0.5	219,159,129,105,81	Germacrone	germacrane-type
21	17.48	$C_{15}H_{24}O$	220.3505	221.1900	0.2	221,203,151,123,109,95,81	Bisacurol	bisabolane-type

* means this component is identified by compared with standard substance.
